# Automatic segmentation and measurement of pressure injuries using deep learning models and a LiDAR camera

**DOI:** 10.1038/s41598-022-26812-9

**Published:** 2023-01-13

**Authors:** Tom J. Liu, Hanwei Wang, Mesakh Christian, Che-Wei Chang, Feipei Lai, Hao-Chih Tai

**Affiliations:** 1 Graduate Institute of Biomedical Electronics and Bioinformatics, National Taiwan University, Taipei, Taiwan; 2 Division of Plastic Surgery, Department of Surgery, Fu Jen Catholic University Hospital, Fu Jen Catholic University, New Taipei City, Taiwan; 3 Department of Electrical Engineering, National Taiwan University, Taipei, Taiwan; 4 Division of Plastic Reconstructive and Aesthetic Surgery, Department of Surgery, Far Eastern Memorial Hospital, New Taipei City, Taiwan; 5 National Taiwan University Hospital and College of Medicine, National Taiwan University, Taipei, Taiwan

**Keywords:** Bacterial infection, Medical imaging, Computational biology and bioinformatics, Computational models, Image processing, Machine learning

## Abstract

Pressure injuries are a common problem resulting in poor prognosis, long-term hospitalization, and increased medical costs in an aging society. This study developed a method to do automatic segmentation and area measurement of pressure injuries using deep learning models and a light detection and ranging (LiDAR) camera. We selected the finest photos of patients with pressure injuries, 528 in total, at National Taiwan University Hospital from 2016 to 2020. The margins of the pressure injuries were labeled by three board-certified plastic surgeons. The labeled photos were trained by Mask R-CNN and U-Net for segmentation. After the segmentation model was constructed, we made an automatic wound area measurement via a LiDAR camera. We conducted a prospective clinical study to test the accuracy of this system. For automatic wound segmentation, the performance of the U-Net (Dice coefficient (DC): 0.8448) was better than Mask R-CNN (DC: 0.5006) in the external validation. In the prospective clinical study, we incorporated the U-Net in our automatic wound area measurement system and got 26.2% mean relative error compared with the traditional manual method. Our segmentation model, U-Net, and area measurement system achieved acceptable accuracy, making them applicable in clinical circumstances.

## Introduction

Pressure injuries, caused by prolonged compression of soft tissue, represent a heavy burden to healthcare systems, affecting millions of patients around the world. The care of pressure sores costs more than $11 billion annually in the United States. Cost of individual patient care ranges from $20,900 to $151,700 per pressure injury^1^.

In this era of the COVID-19 pandemic, transporting patients to receive treatment at medical facilities increases their risk of contracting COVID-19. Telemedicine for the wound care of these patients may thus be helpful in reducing medical costs, avoidance of infection sources and making treatment more efficient. In telemedicine, accurate measurement of the wound area is critical to the evaluation and management of chronic wounds to monitor the wound healing trajectory and to determine future interventions. However, manual measurement is time-consuming and inconvenient for first-line caregivers. To build a system that can automatically measure the wound area, we need to do wound segmentation first.

Previous studies on wound segmentation can be roughly categorized into two groups: traditional methods and deep learning methods. Studies in the first group apply manual feature extraction with traditional algorithms, such as K-means clustering, edge detection, thresholding, region growing, etc^2–6^. These methods suffer from the following limitations: (1) as in many computer vision systems, the handcrafted features are affected by the environment and image resolution; (2) they are not immune to severe pathologies and rare cases, which are very impractical for a clinical circumstance.

Unlike traditional methods, deep learning methods based on the structure of neural networks in the human brain have shown promising performance in medical image processing^7^. Since the successes which AlexNet^8^ achieved in the 2012 ImageNet large scale visual recognition challenge, development of applications of deep learning in the domain of computer vision has begun using deep convolutional neural networks (CNNs). The CNNs extract the features and determine their importance when training. One successful architecture of CNN for segmentation is fully convolutional neural networks (FCN)^9^. An FCN comprises only convolutional layers without a fully connected layer. Several FCN-based models have been proposed to solve the problem of wound segmentation. For example, Wang et al. proposed the for vanilla FCN architecture for wound segmentation^10^. However, the Dice coefficient of the segmentation was only 64.2%. Goyal et al. proposed the FCN-16 architecture on wound images^11^. They were able to achieve a Dice coefficient of 79.4% on their dataset. However, the network’s segmentation accuracy is limited when distinguishing small wounds and wounds with irregular borders as it tends to draw smooth contours. Liu et al. proposed a new FCN architecture that replaces the decoder of the vanilla FCN with a skip-layer concatenation up-sampled with bilinear interpolation^12^. A Dice accuracy of 91.6% was achieved on their dataset of 950 images taken under an uncontrolled lighting environment with a complex background. However, images in their dataset were semi-automatically annotated using a watershed algorithm. Wang et al. proposed a novel convolutional framework based on MobileNetV2 and connected component labelling to segment wound regions from natural images and achieved a Dice coefficient of 90.47%^13^. Chang et al. tested five deep learning models, U-Net, DeeplabV3, PsPNet, FPN, and Mask R-CNN, based on superpixel segmentation assisted labeling to segment pressure ulcers and DeeplabV3 had the best performance with an accuracy of 0.9925^14^. However, no external validation was conducted. Deep learning for wound segmentation is now a reliable technique and some studies have achieved comprehensive results.

After automatic segmentation of the wound is performed, another hardware device is needed to get depth information in order to calculate the length and area of the wound. LiDAR (light detection and ranging) is a technique for determining ranges (variable distance) by targeting an object with a laser and measuring the time for the reflected light to return to the receiver. Using cameras and LiDAR devices together, we can obtain 3D and 2D information, and theoretically get the length and area of the objects in the real world.

The objective of our study focused on: (1) conducting a fully automatic segmentation model with high accuracy and (2) conducting automatic wound area measurement with a camera with LiDAR.

## Methods

### Data collection and labeling for deep learning training

To train the segmentation model, we needed a large sample of labeled clinical photos. We retrospectively reviewed the medical records of patients who were diagnosed with pressure injuries from 2016 to 2020 in National Taiwan University Hospital. Of the 1,038 photos collected from the records, we eliminated those which were blurred, overexposed, underexposed, obscured, or which contained too many identifiable objects or features other than the wound. Finally, we selected a total of 528 photos of pressure injuries for inclusion. We used the 327 photos from 2016 to 2019 for training and internal validation and the 201 photos from 2019 to 2020 for external validation. After the system of automatic area measurement was built, we performed prospective study to validate its accuracy. The details will be described in the later section. We confirmed that all methods in our study were performed in accordance with the relevant guidelines and regulations and the study was approved by National Taiwan University Hospital’s ethics committee (202005032RINB). All the patient’s name and any other identifying information were removed before analysis.

Three board-certified plastic surgeons were recruited to label the margins of the pressure injuries without regard to staging using the labeling tool “LabelMe” and save them as json files. The LabelMe annotator tool, an open-source program by Kentaro Wada, can be used to annotate polygonal, rectangular, circular, and pointed shapes^15^. All photos were co-labeled to yield a single consensus result.

Since the photos of pressure injuries were collected from various medical records, their sizes were not uniform. All labeled images were re-sized to 512*512 pixels. We applied two deep learning architectures, U-Net and Mask R-CNN, in combination with a ResNet101 backbone to segment these images.

### Semantic segmentation: U-Net

U-Net^16^, proposed by Olaf et al., is a type of convolutional network with U-shaped architecture to extract and preserve features for the object segmentation task. First designed to process biomedical images, U-Net has the capacity to localize and distinguish segmentation by classifying each pixel to each class so that its input and output result size are identical.

The major parts of the U-Net architecture are the left path called the contracting (down-sampling) path, and the right path, which is constituted by transposed 2d convolution of expanding layers (up-sampling), and the skip-connections that share feature maps from the down-sampling path to the up-sampling path. The down-sampling path, repeated application of CNNs, each consists of two 3 × 3 convolutions, followed by a rectified linear unit (RELU) activation and a 2 × 2 max pooling of feature channels, especially used to extract features from an image as the spatial information decreases. On the other hand, the up-sampling path, each step consisting of a 2 × 2 convolution (“up-convolution”), combines the features and spatial information through a sequence of up-convolutions that halves the number of feature channels and merges with the feature maps from the down-sampling path to classify each pixel. Furthermore, to address the loss of spatial information that occurs in the down-sampling path, the authors introduced skip-connections. A skip-connection’s main function is to deliver the higher resolution feature maps from the down-sampling path to the up-sampling path so that the up-sampling path can reconstruct the information that decreased during the down-sampling path and learn better feature representations with following convolutions.

In our study, we trained our model using standard augmentation such as rotations, shifts, scale, gaussian blur, and contrast normalization. We trained our U-Net with replacement of the convolutions path with a ResNet-101 backbone^17^, which can explore and learn more features from the data. Then, the networks can be initialized using pre-trained model weights derived from large-scale object detection, segmentation and captioning datasets such as ImageNet^18^, in which there are more than 14 million labeled photos. The standard Dice loss was chosen as the loss function. The formula is given by:

1 $$L\left(TP,FP,FN\right)=1- \frac{2TP+\epsilon }{2TP+FP+FN+\epsilon }.$$

The ∈ term is used to avoid the issue of dividing by 0 when precision and recall are empty.

### Instance segmentation: mask R-CNN

Mask R-CNN^19^ is a state-of-art deep learning model developed by the Facebook AI research team (FAIR) in April 2017. Mask R-CNN is an extended version of Faster R-CNN, which solves instance segmentation problems, and is able to distinguish objects within the same class as an individual instance.

Mask R-CNN separates the mask predictions independently to another branch in parallel with a combination of bounding box prediction branches. It consists of two stages. In the first stage the Regional Proposal Network (RPN) generates a regional proposal of the objects in an image. In the second stage the binary mask classifier, whose function is to do classification, then improves the proposed bounding box from RPN and adds mask prediction to the object.

In our implementation of Mask R-CNN, we trained our model using a ResNet-101 backbone with weights from the pre-trained Microsoft COCO (common objects in context) database^20^, which is a large-scale object detection, segmentation, and captioning dataset. Mask R-CNN uses a multi-task loss function given by L = Lclass + Lbox + Lmask. The Lclass component contains the RPN class loss (failure of the Region Proposal Network to separate object prediction from background) added to the Mask R-CNN class loss (failure of the Mask R-CNN object classification). The Lbox component contains the RPN bounding box loss (failure of object localization or bounding by RPN) added to the Mask R-CNN bounding box loss (failure of object localization or bounding by Mask R-CNN). The last component Lmask loss constitutes the failure of Mask R-CNN object mask segmentation.

### LiDAR

LiDAR (light detection and ranging) technology, implanted in a high-level smartphone or tablet such as an iPhone 12 Pro or iPad Pro or more advanced type, is a method for determining ranges (variable distance) by targeting an object with a laser and measuring the time for the reflected light to return to the receiver.

Initially the camera takes a 2-Dimensional (2D) image, and the LiDAR sensor takes depth information of pressure injuries in the scene we want to detect. Although the center point of the camera and the LiDAR module is different and the distance between these two points is about 1.5-cm, Apple Inc. has already done the point matching calibration between the camera and the LiDAR for users. Therefore, users do not have to worry about the point matching of the 2D image from the camera and the depth image from the LiDAR sensor.

**Figure 1:**
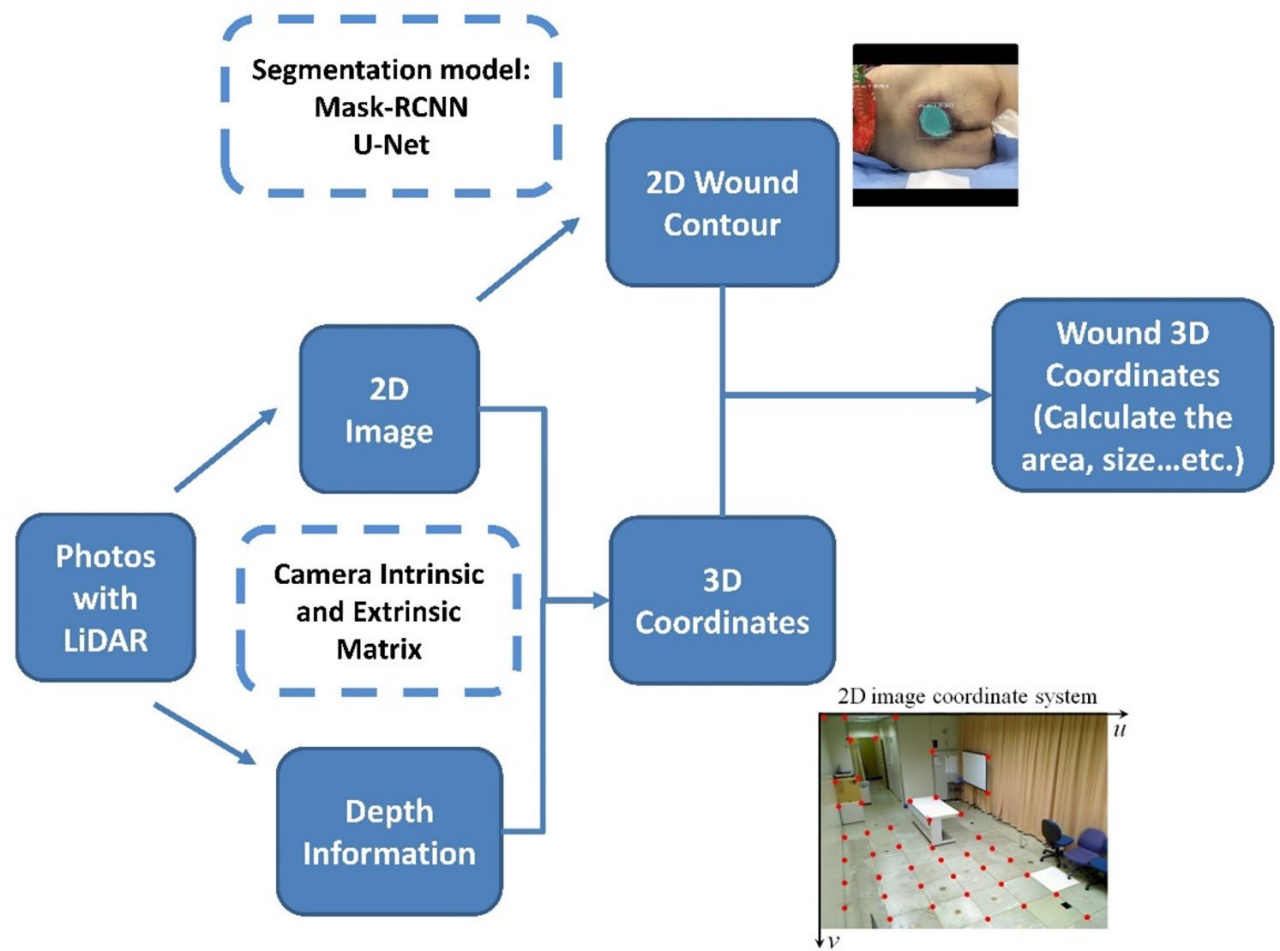
The algorithm for the automatic area measurement of pressure injuries.

Thus, we analyze the 2D image using segmentation models which we built previously and get the border of the wound in in 2D coordinates. Using the 2D image and depth information we can convert 2D coordinates into 3-Dimensional (3D) wound coordinates using the camera’s intrinsic and extrinsic matrixes. Finally, we use the 3D coordinates of the wound contour to do the measurement of the wound area (Fig. 1).

### Real-world coordinate conversion

With the 2D images from the camera and the depth data from LiDAR, we can convert the information into 3D real world coordinates using (2), where *m*_image_ is the 2D coordinate vector [*u v l*]^T^ of the image and *M*_world_ is the 3D coordinate vector [*x y z l*]^T^ of the real world wound, *K* is the camera intrinsic matrix, and [*R*|*t*] is the camera extrinsic matrix.

2 $${m}_{image}=K\left[R|t\right]{M}_{world} .$$

The camera intrinsic matrix allows you to transform 3D camera coordinates to 2D image coordinates on an image plane using the pinhole camera model by using (3). The values *f*_x_ and *f*_y_ are the pixel focal lengths; *o*_x_ and *o*_y_ are offsets of the principal point from the top-left corner of the image frame. Since *m*_image_ is known and the *z* value of *M*_camera_ can be replaced by the depth information acquired by LiDAR, the remaining *x, y* values of *M*_camera_ can be resolved.

3 $${m}_{image}=K{ \,M}_{camera},K = \left[\begin{array}{ccc}{f}_{x}& 0& {o}_{x}\\ 0& {f}_{y}& {o}_{y}\\ 0& 0& 1\end{array}\right].$$

The camera extrinsic matrix [*R*|*t*] is a matrix relating to a camera's position and orientation to a world or scene coordinate system, which is a matrix concatenation of a 3*3 rotation matrix R and 3*1 column vector translation *t*. Once we obtain *M*_camera_, we can use the camera extrinsic matrix to transfer from 3D camera coordinates into 3D real world coordinates by using (4)

4 $${M}_{camera}=\left[R|t\right]{M}_{world}.$$

### Area measurement by Heron’s formula

All the 3D coordinates of the wound’s border can be projected onto a *Plane A* with the shortest average distance with the formula:

5 $$Plane \,A=f\left(x,y\right)=z=ax+by+c.$$

Given multiple coordinates (*x*_*i*_*, y*_*i*_*, z*_*i*_) of the wound’s border, the variables *a, b, c* can be found by the following steps:

a. Assume that all the *x, y* coordinates form the first two columns of *Matrix A*:

6 $$Matrix\, A= \left[\begin{array}{c}\begin{array}{ccc}{x}_{1}& {y}_{2}& 1\\ {x}_{2}& {y}_{2}& 1\\ {x}_{3}& {y}_{3}& 1\end{array}\\ \vdots \\ \begin{array}{ccc}{x}_{n}& {y}_{n}& 1\end{array}\end{array}\right].$$

b. Assume the variables *a, b, c* we want to solve for constitute *vector x*:

7 $$vector \,x= \left[\begin{array}{c}a\\ b\\ c\end{array}\right].$$

c. Assume all the *z* coordinates form *vector B*:

8 $$vector \,B= \left[\begin{array}{c}{z}_{1}\\ {z}_{2}\\ \begin{array}{c}{z}_{3}\\ \begin{array}{c}\vdots \\ {z}_{n}\end{array}\end{array}\end{array}\right].$$

Because *Matrix A* and *vector B* are given, combining (6), (7), (8), we obtain:

9 $$B=Ax.$$

To solve for *vector x*, in other words the coefficients, is reduced to solving the equations of multiple linear regression, or the regression plane in our case.

After *Plane A* is found, the projection points onto *Plane A* of all the wound’s border coordinates (*x’*_*i*_*, y’*_*i*_*, z’*_*i*_) can be easily found. Then the projection points can be used to estimate the area of the wound by Heron’s formula^21^ and trigonometric functions. The equation is:

10 $$a= \frac{1}{2\mathrm{cos}\theta }\left(\left|\begin{array}{cc}{x^{\prime}}_{n}& {x^{\prime}}_{1}\\ {y^{\prime}}_{n}& {y^{\prime}}_{1}\end{array}\right|+\left|\begin{array}{cc}{x^{\prime}}_{1}& {x^{\prime}}_{2}\\ {y^{\prime}}_{1}& {y^{\prime}}_{2}\end{array}\right|+\cdots\text{+}\left|\begin{array}{cc}{x^{\prime}}_{n-2}& {x^{\prime}}_{n-1}\\ {y^{\prime}}_{n-2}& {y^{\prime}}_{n-1}\end{array}\right|+\left|\begin{array}{cc}{x^{\prime}}_{n-1}& {x^{\prime}}_{n}\\ {y^{\prime}}_{n-1}& {y^{\prime}}_{n}\end{array}\right|\right),$$

where *a* means the area estimation, the coordinates (*x’*_1_, *y’*_1_), …, (*x’*_n-2_, *y’*_n-2_), (*x’*_n-1_, *y’*_n-1_), (*x’*_n_, *y’*_n_) are the projection points on *Plane A* of all the wound’s border points. *θ* is the angle between *Plane A* and the x–y plane.

### Prospective validation of the automatic wound area measurement

To validate the accuracy and reliability of the automatic wound area measurement, we conducted a prospective clinical test. From June 2021 to January 2022, we measured the area of pressure injuries of patients who visited our outpatient department (OPD) at National Taiwan University Hospital (NTUH) using the traditional manual method as well as our automatic area measurement system. The informed consent was obtained from all patients before we performed the wound area measurement. When 20 pressure injuries had been collected, the study was completed and the statistical analysis was begun. For each patient a photo was taken that was clear enough to let the system work and there was one wound per image. All the patient’s name and any other identifying information were removed before analysis.

**Figure 2:**
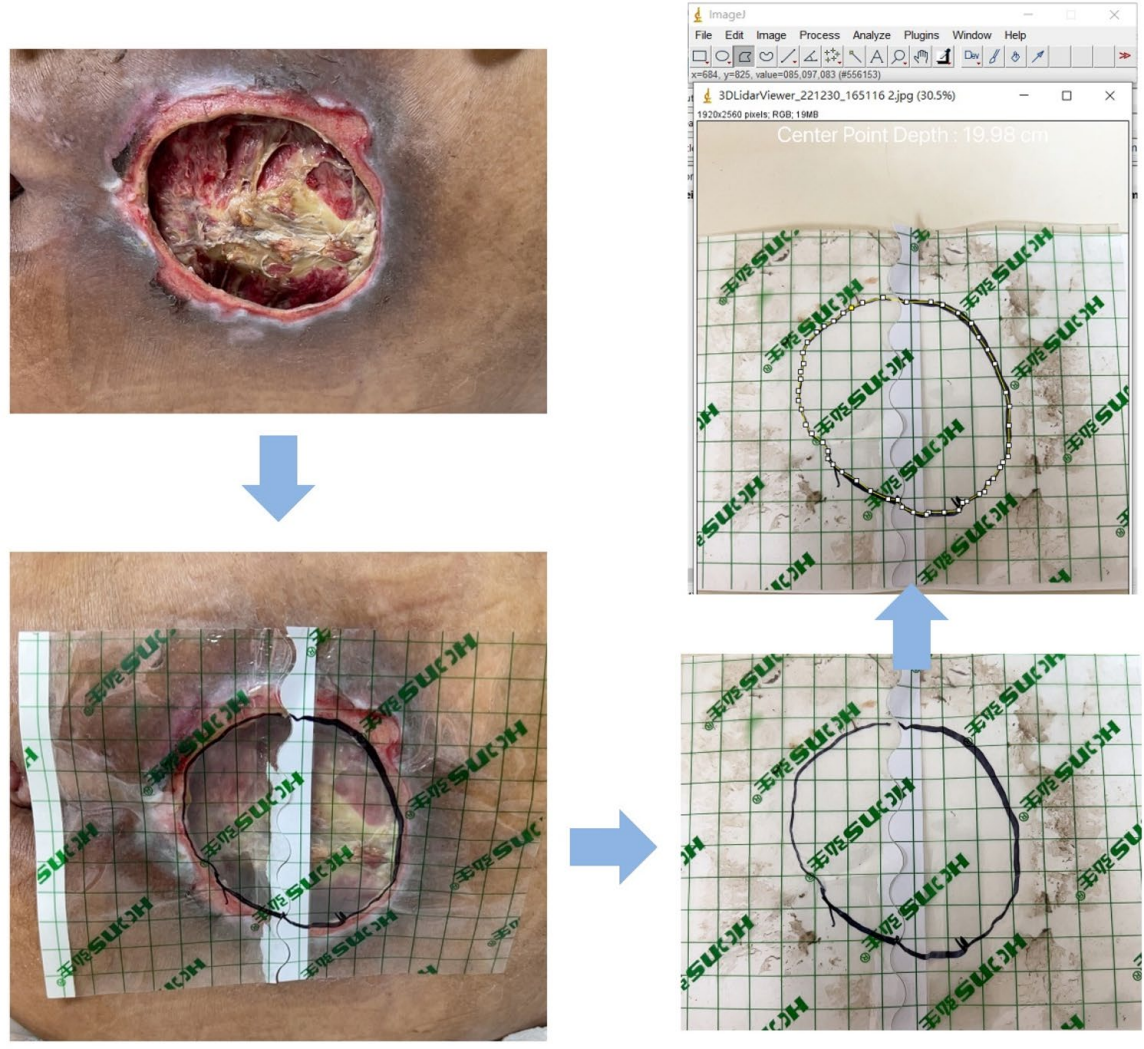
The traditional manual method for the measurement of wound area. (Upper right) Final measurement by the software ImageJ.

The traditional method is to use scaled transparent film to cover the wound and then a marker pen to delineate the border. The area and width in the delineated contour were checked by two board-certified plastic surgeons and were then measured by the software ImageJ (National Institutes of Health, USA), which is an open-source, Java-based reliable imaging tool^22^ (Fig. 2).

### Statistical analysis

#### The performance of automatic segmentation

The Dice coefficient (DC) and the Intersection over Union (IoU) are two common metrics used to assess segmentation performance, whereas the precision, recall and accuracy are the common metrics of assessing classification performance. DC is twice the area of the intersection of the ground truth and prediction divided by the sum of their areas. It is given by:

11 $$DC=\frac{2\left|Area\left(Predict\right) \cap Area(Ground\, truth)\right|}{\left|Area\left(predict\right)\right|+\left|Area(Ground \,truth)\right| }\,\,\mathrm{ or }\,\,\frac{2TP}{2TP+FP+FN},$$

where TP (true positive) denotes the number of correctly classified pressure injury (PI) pixels; FP (false positive) denotes the number of mistakenly classified PI pixels; FN (false negative) denotes the number of mistakenly classified non-PI pixels.

The intersection over union (IoU) denotes the area of the intersection of the ground truth and prediction divided by the area of their union. It is given by:

12 $$IoU=\frac{\left|Area\left(Predict\right) \cap Area(Ground \,truth)\right|}{\left|Area\left(predict\right) \cup Area(Ground\, truth)\right|} \,\,or \,\,\frac{TP}{TP+FP+FN} .$$

Precision is defined as the ratio of the number of PI pixels correctly classified to the number of all predicted pixels. It is also called positive predictive value and given by:

13 $$Precision= \frac{TP}{TP+FP} .$$

Recall is defined as the ratio of the number of PI pixels that are correctly classified to the total number of PI pixels. It is also called sensitivity and given by:

14 $$Recall=\frac{TP}{TP+FN} .$$

Accuracy denotes the percentage of correctly classified pixels. It is given by:

15 $$Accuracy=\frac{TP+TN}{TP+FP+TN+FN},$$

where TN (true negative) denotes the number of correctly classified non-PI pixels.

#### The performance of automatic wound area measurement

To evaluate the performance of the automatic wound area measurement, we calculated the mean relative error (MRE) and the standard deviation (SD,$\sigma$) of MRE of each of the two models, U-Net and Mask R-CNN. The MRE is given by:

16 $$Mean \,Relative \,Error \,\left(MRE\right)= \frac{1}{n} \sum_{i=0}^{n}\frac{\left|{A}_{i}^{*}-{A}_{i}\right|}{{A}_{i}},$$

where *A*_*i*_ denotes the measurement of the area by the traditional method and A^*^_i_ denotes the measurement of the area by the automatic method. The standard deviation (SD,$\sigma$) is given by:

17 $$Standard \,deviation \,\left(SD, \sigma \right)=\sqrt{\frac{\sum_{i=0}^{n}{({x}_{i}-\overline{x })}^{2}}{n-1}},$$

where *x*_*i*_ denotes the RE of the *i*th (*i* = 1 ~ 20) automatic measurement, and $\overline{x }$ denotes the MRE.

## Results

### The performance of automatic wound segmentation

#### Internal validation

Most of the photos contained one wound per image in our training set. The average number of wounds per image was 1.14. Both U-Net and Mask R-CNN with a ResNet101 backbone performed well on the internal validation. We trained both our models for 1,000 epochs with a learning rate of 0.0001. On the internal validation task, Mask R-CNN performed better than U-Net (DC: 0.9464 versus 0.9441; IoU: 0.9337 versus 0.8982). Other statistics are detailed in Table 1.

**Table 1:** The performance of the segmentation models.

Models	U-Net		Mask R-CNN	
	Internal validation	External validation	Internal validation	External validation
Mean DC^a^	0.9441	0.8488	0.9464	0.5006
Mean IoU^b^	0.8982	0.7773	0.9337	0.4604
Mean precision	0.9559	0.8756	0.9739	0.5017
Mean recall	0.9361	0.8639	0.9204	0.5614
Mean accuracy	0.9899	0.9807	0.9613	0.4972

^a^DC: Dice coefficient.

^b^IoU: Intersection over union.

#### External validation

External validation is the testing of the original model on a set of new data to determine whether the model works to a satisfactory degree and whether overfitting has occurred. We found that U-Net and Mask R-CNN both performed worse on external validation and that the performance of U-Net was better than Mask R-CNN (DC: 0.8448 versus 0.5006; IoU: 0.7773 versus 0.4604). Other statistics are detailed in Table 1.

### The performance of automatic wound area measurement

**Figure 3:**
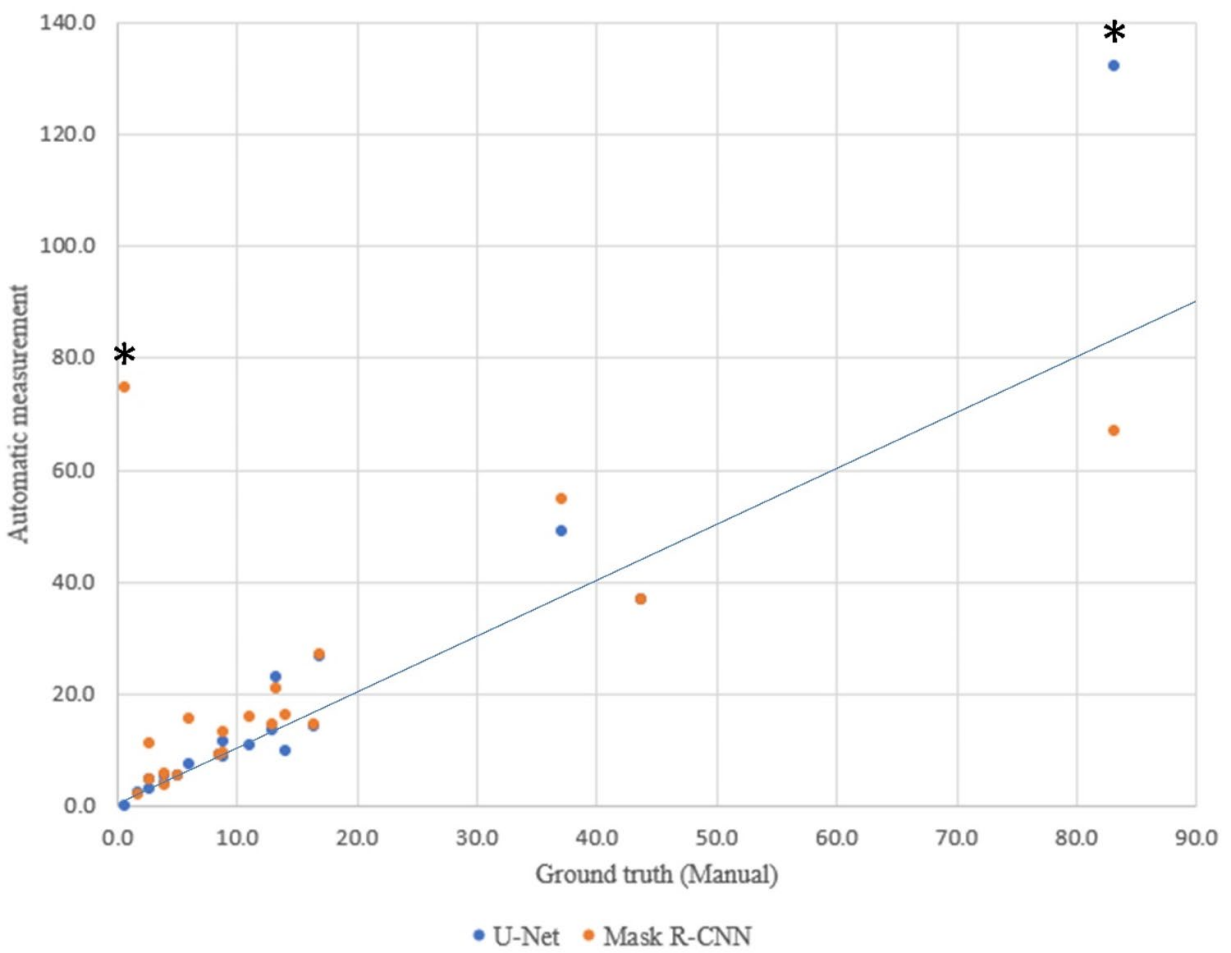
Comparison of manual and automatic wound area measurements. * The outliers.

The comparison of the automatic wound area measurements by U-Net and Mask R-CNN regarding the traditional manual method is shown in Table 2 and Fig. 3. We noted there were two outliers: wound No. 16 and No. 20. We checked the segmentation processes to ascertain what caused these outliers. Further discussion of the outliers is detailed in the discussion section.

**Table 2:** The comparison of manual and automatic wound area measurements.

Ulcer no.	Manual	U-Net		Mask R-CNN	
Area	cm^2^	cm^2^	RE (%)	cm^2^	RE (%)
1	1.8	2.5	40.7	2.1	19.2
2	3.9	4.1	3.6	3.9	0.8
3	16.4	14.1	13.6	14.6	11.0
4	2.7	3.2	22.3	4.7	76.6
5	14.1	9.8	30.3	16.3	16.1
6	3.9	5.5	42.4	5.7	46.5
7	43.8	37.0	15.5	36.8	16.1
8	8.8	8.7	1.4	9.4	6.5
9	8.6	9.3	8.8	9.3	8.4
10	13.4	23.0	72.3	21.0	57.1
11	17.0	26.7	56.9	27.1	59.4
12	5.1	5.5	7.1	5.4	5.8
13	8.9	11.4	27.5	13.3	48.7
14	2.7	4.6	72.9	11.3	320.3
15	6.1	7.6	25.6	15.8	159.2
*16	0.7	0.0	100.0	74.8	10,345.5
17	13.0	13.6	4.5	14.5	11.6
18	11.0	10.9	0.5	15.9	44.2
19	37.1	49.1	32.2	54.7	47.3
*20	83.2	132.0	58.7	67.0	19.5
MRE			31.8		566
SD of MRE			0.266		21.9
MRE (outliers removed)			26.2		53
SD of MRE (outliers removed)			0.23		0.75

*RE* Relative error, *MRE* Mean relative error, *SD* Standard deviation.

*The outliers.

The MREs of U-Net and Mask R-CNN were 31.8% and 566%, respectively. After excluding the outliers, the MREs of U-Net and Mask R-CNN were 26.2% and 53%. The SD of the relative error of U-Net and Mask R-CNN were 0.23 and 0.75. The performance of U-Net was better than Mask R-CNN and was consistent with the result of the external validation of the automatic segmentation.

## Discussion

### Principal results

#### Automatic wound segmentation

U-Net and Mask R-CNN were chosen as our models for segmentation of pressure injuries because they are both the classic CNN models for segmentation. U-Net provides semantic segmentation and is the most popular model for segmentation of the biomedical images^23^. U-Net has been utilized for different kinds of medical images, such as CT^24^, MRI^25^, PET^26^ scans of lesions from different organs and microscopy images^27,28^. Mask R-CNN provides instance segmentation^19^ and has been utilized for different kinds of medical images as well, such as MRI scans of knee^29^, PET scans of lung^30^, ultrasonography of breast^31^ and microscopy images^28^.

In our study, Mask R-CNN, on internal validation, was a little better than U-Net on pressure injuries segmentation (IoU: 0.9337 versus 0.8982). However, on external validation, Mask R-CNN performed very poorly (IoU: 0.4604) while the performance of U-Net was relatively acceptable (IoU: 0.7773).

**Figure 4:**
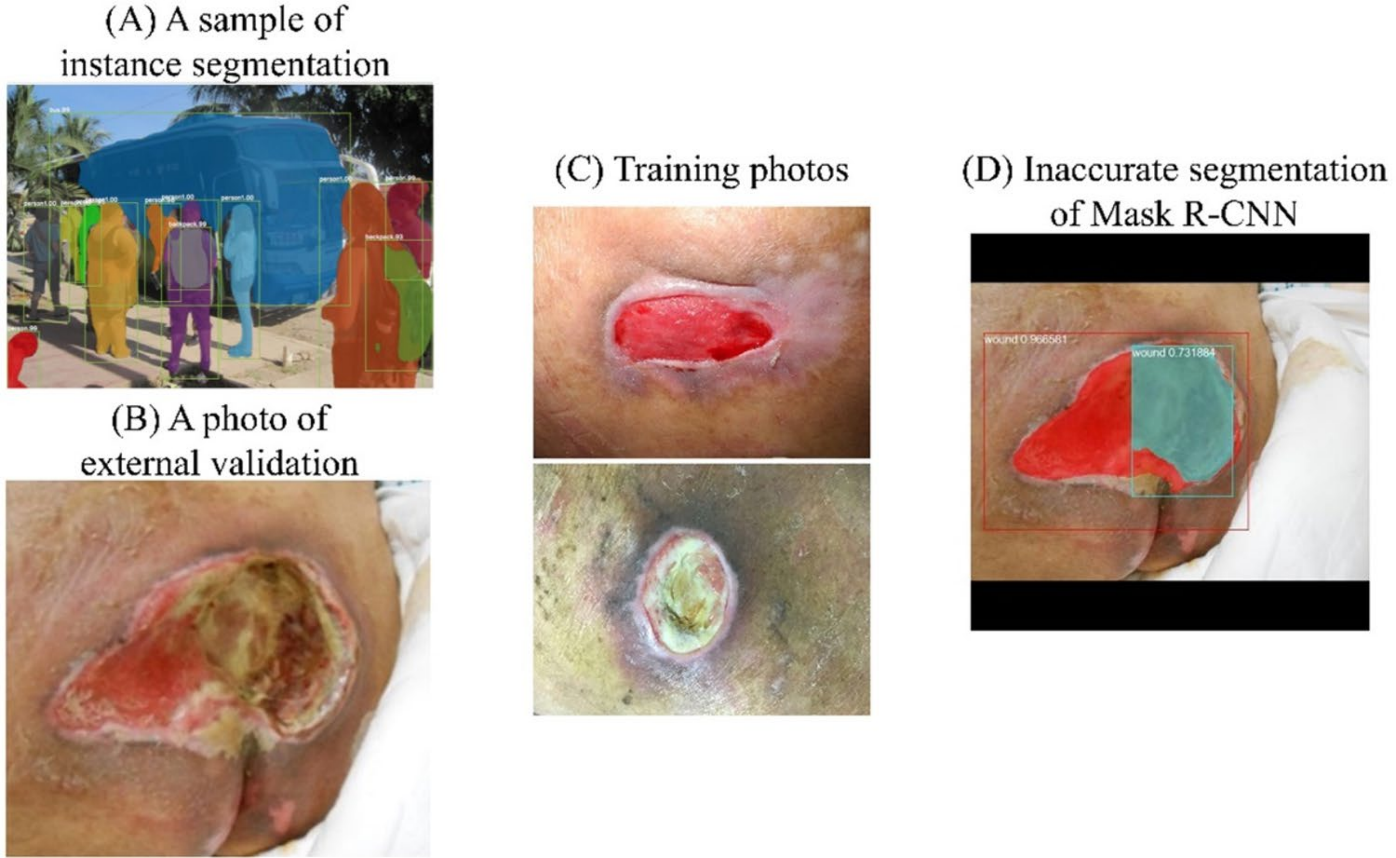
The “object-in-object” phenomenon. (**A**) The instance segmentation is good for discrimination of different people overlapping with each other. (This sample image was the prediction of Mask R-CNN on COCO dataset^20^.) (**B**) However, one wound may contain multiple textures. (**C**) In the training set, we could identify different textures in these two solitary wounds. (**D**) Mask-RCNN inaccurately performed segmentation, identifying a second smaller “wound” with different texture (blue) within the bigger wound (red). However, in actuality, there was only one wound.

**Figure 5:**
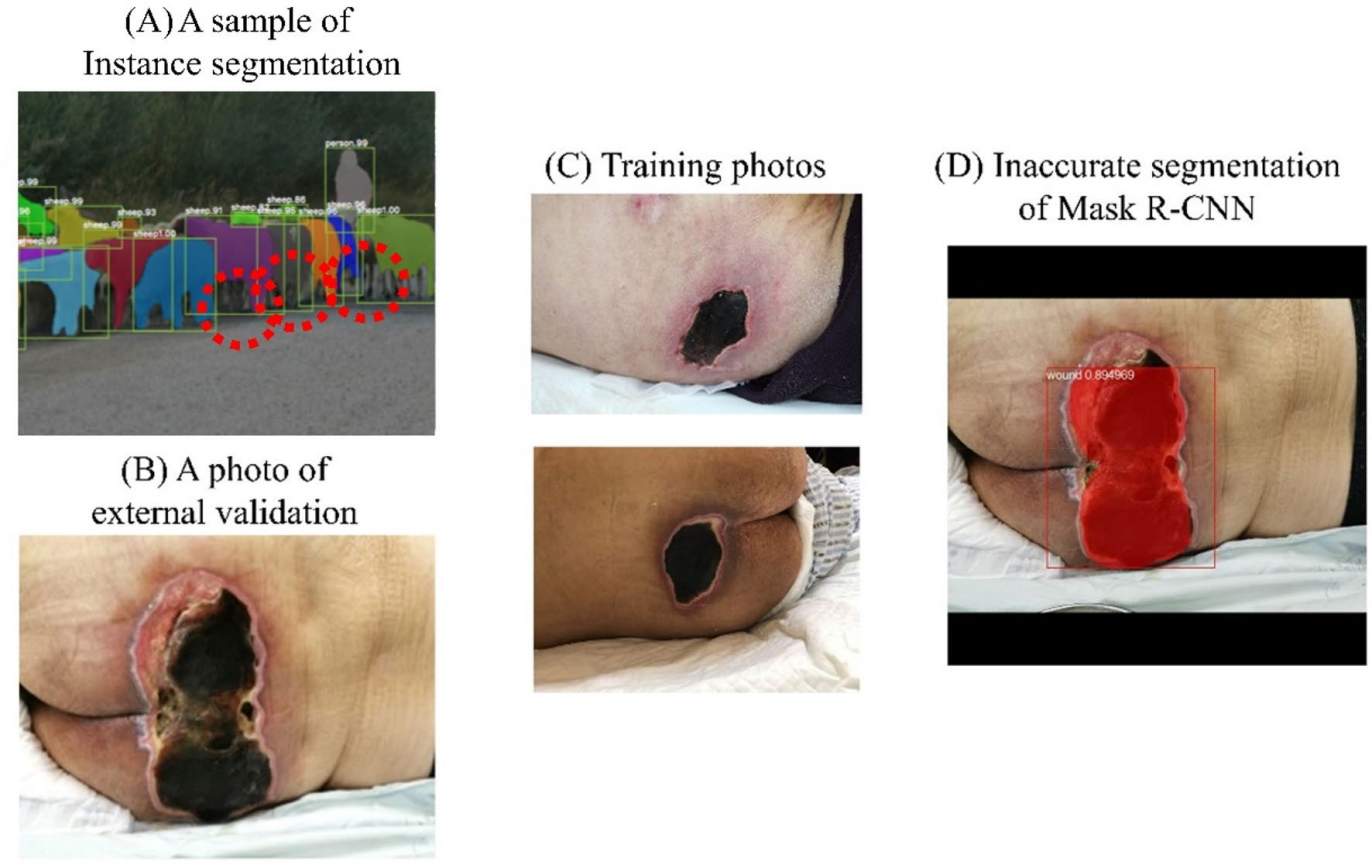
The “cut-off-head” phenomenon. (**A**) Due to the architecture of Mask R-CNN, the instance segmentation is subject to getting “trapped” by an earlier identified ROI (region of interest). The red dotted circles indicate that the feet of the sheep were not accurately segmented. (This sample image was the prediction of Mask R-CNN on COCO dataset^20^.) (**B**) A single wound may have different textures with an irregular shape, making it vulnerable to being inaccurately segmented as multiple wounds. (**C**) In the training set, we found some single texture wounds with regular circular shapes. (**D**) Mask-RCNN inaccurately segmented the more complex wound by “missing” the upper part of the wound.

A plausible explanation for this, supported by the following considerations, is that Mask R-CNN was overfitting in the training set while U-Net was not. First, U-Net is known for achieving good performance on biomedical image segmentation when trained with limited datasets. In a study by the inventors of U-Net, Ronneberger et al. trained U-Net with a dataset of only 30 images, combined with data augmentation, for which they won the International Symposium on Biomedical Imaging (ISBI) competition in 2015^16^. Second, pressure injuries (PI) are staged into four types, based on color, hue and texture, and are of irregular shape and different sizes. Instance segmentation models such as Mask R-CNN are required to do object detection first and then mask segmentation. They must take into consideration the loss function components from estimating the bounding box and class, not just the mask. The weights of the bounding box and class components are calculated prior to the weight of the mask component in order to get accurate instance location. In other words, they are not only trained to distinguish the PIs from background but also to distinguish some classes of PIs from others. This intention, combined with the nature of an individual PI, can result in the model finding other PIs in some specific PI or overlook some part of an individual PI, especially when the training dataset is limited. These two phenomena, which we call “object-in-object” and “cut-off-head,” are described in Figs. 4 and 5. On the other hand, U-Net, a kind of semantic augmentation, does not have this kind of problem. However, these two phenomena may be caused by a limited training set. We can conclude that when dealing with a limited training set of biomedical images, U-Net is better than Mask R-CNN.

#### Automatic wound area measurement

**Figure 6:**
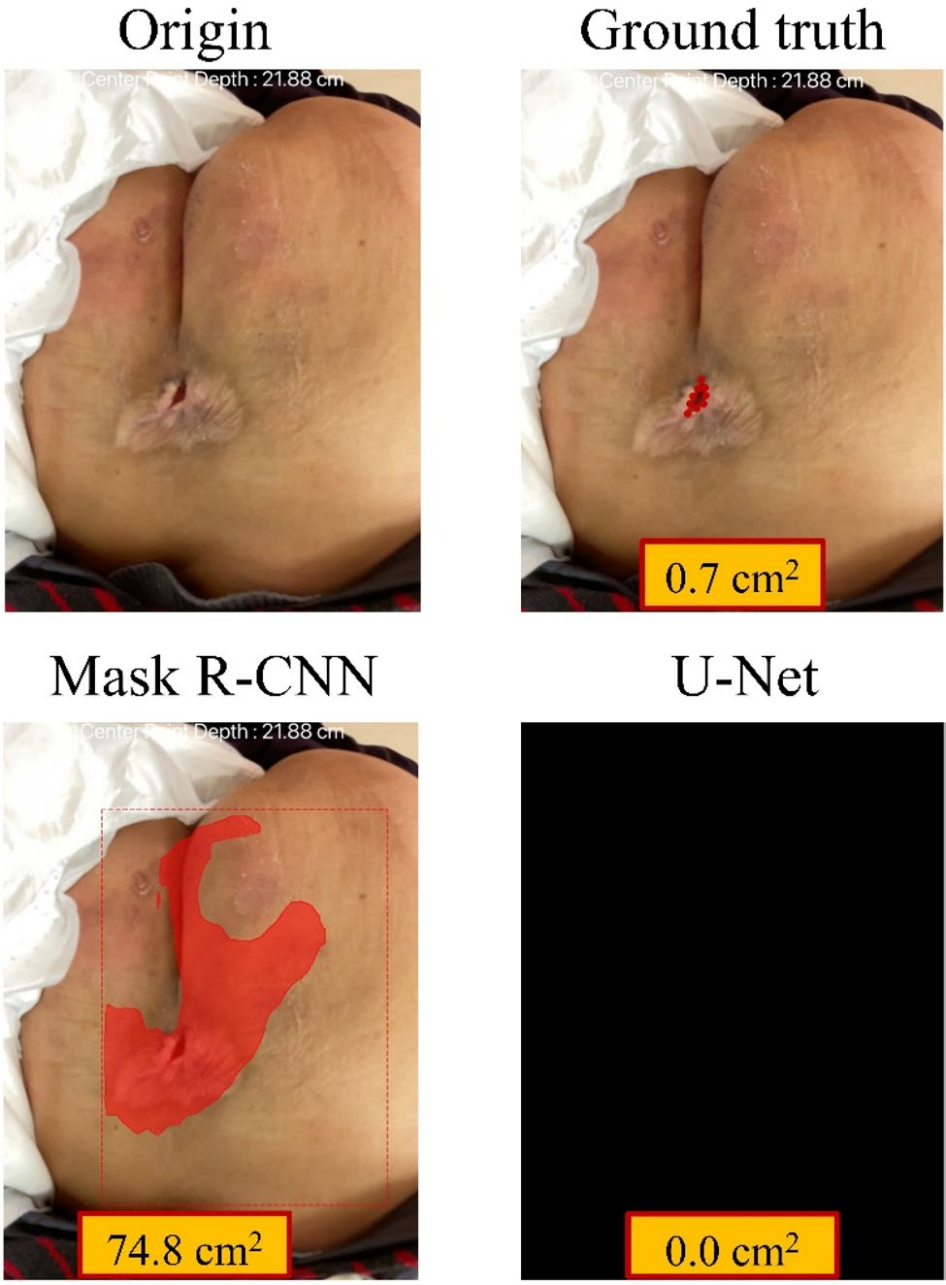
The segmentation process of wound No. 16.

**Figure 7:**
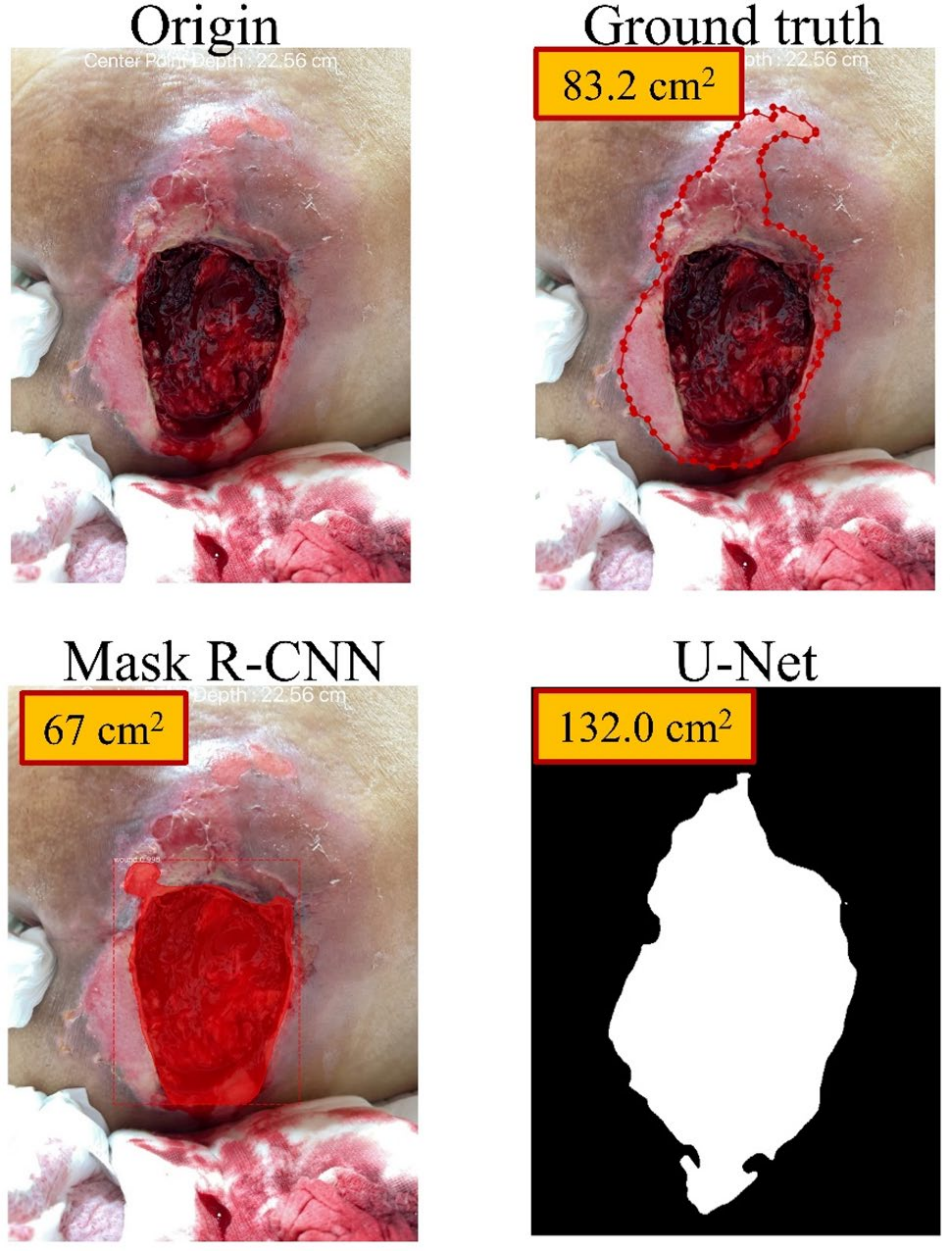
The segmentation process of wound No. 20.

U-Net performed better at automatic wound area measurement than Mask R-CNN (MRE: 19.14% versus 565.98%), consistent with the results of the external validation of the segmentation. We noted there were two outliers: wounds no. 16 and no. 20 (Figs. 6 and 7). In the segmentation process of wound no.16, we found that the proportion of the wound to the whole picture was too small to successfully do the segmentation. Therefore, we have added an instruction to our system that the wound must be centered and fill at least 20% of the whole picture and instructs the user to center the camera and move the camera closer to the wound when it is not.

In the segmentation process of wound no. 20 (Fig. 7), we encountered two different segmentation problems. The original photo showed two types of wound texture: one is pink and shallow, and the other is dark red and deep. As defined, they were all considered as one wound, but Mask R-CNN only segmented the dark red one. U-Net successfully segmented these two types of wound texture as one wound; however, due to too much blood and gauze in the picture, a condition that may be due to the photo having been taken just after surgery, U-Net inaccurately segmented some of the area with the blood and gauze as part of the wound area. Consequently, Mask R-CNN underestimated the area while U-Net overestimated it. We considered this situation as an outlier because if the picture had been clean with only wound content in the picture, U-Net would not have overestimated the area. Therefore, we have added an instruction for users of our system that the photo should be taken with a clean background and with no distracting features in the picture. Another approach to improving the outcome of our system in future work is to collect more photos in the training set with a messy background in order to train the model to better distinguish the wound from other features.

Another issue is that why we used the “the area” of the wound rather than its “volume.” The reason is that we could not actually get the ground truth of the volume by the traditional method, although the volume of the wound better reflects the severity of the wound because the depth of the wound is important as well. If we can prove the LiDAR technology can get accurate 3D coordinates by checking the area of the wounds compared with traditional manual method, we can indirectly prove that we can get accurate depth and volume of the wound via mathematics by LiDAR technology.

There are some previously published studies proposing different deep learning algorithms to do the wound segmentation as we mentioned before^10–14,32–34^. The types of deep learning, their performance, and other details are listed in Table 3. Although some of them achieved good performance on segmentation, these studies did not propose methods for the wound area measurement.

**Table 3:** Comparison of different deep learning methods for wound segmentation in different studies.

Authors (year)	The best model (Total number of trained models)	Type of ulcer	Database (number)	Internal validation (The best model)		External validation (The best model)		Comments
				DC	IoU	DC	IoU	
Our study, 2022	U-Net (2)	PU	Internal:327 External:201	0.944	0.898	0.849	0.777	1. Combined with two state-of-art models 2. Combined with automatic wound area measurement
Wang et al.^10^	ConvNet (2)	Chronic ulcers	Internal:500 External:150	N/A	N/A	N/A	0.473	Compared with machine learning (SVM)
Goyal et al.^11^	FCN-16 s (4)	DFU	Internal:480 External:120	N/A	N/A	0.794	N/A	Compared with FCN with different layers
Liu et al.^12^	MobileNet-FCN16 (3)	Ulcers	Internal: 900	0.917	0.846	N/A	N/A	1. Lacks external validation 2. Using watershed algorithm
Wang et al.^13^	MobileNetV2 with CCL (6)	Foot ulcers	Internal: 1109 External: Medetec dataset	0.905	N/A	0.945	N/A	Requiring post-segmentation process
Chang et al.^14^	DeepLabV3 (5)	PU	Internal: 2893	0.989	0.978	N/A	N/A	1. Combined with tissue segmentation 2. Lacks external validation
García-Zapirain et al.^30^	3D CNN	PU	Internal: 193	0.92	N/A	N/A	N/A	Lacks external validation
Ohura et al.^31^	U-Net (4)	PU → DFU + VLU	Internal: 400 (PU) External:20 (DFU); 20 (VLU)	0.936	N/A	0.850	N/A	Using PU-trained model to do segmentation of DFU and VLU
Zahia et al.^32^	CNN	PU	22	N/A	N/A	N/A	N/A	Only for tissue classification

*DC* Dice coefficient, *IoU* Intersection over union, *PU* Pressure ulcer, *DFU* Diabetic foot ulcers, *VLU* Venous leg ulcer, *CNN* Convolutional neural network, *U-Net* Mask R-CNN; SVM; ConvNet; *FCN* Fully convolutional networks, *SVM* Support vector machine, *CCL* Connected component labelling.

Previous studies have also addressed wound area measurement. Ahmad Fauzi et al.^3^ proposed wound area measurement by a “label card” beside the wound with a traditional segmentation model based on a Red-Yellow-Black-White (RYKW) probability map combined with a modified hue-saturation-value (HSV) model. They achieved an accuracy about 75.1%. Wang et al.^35^ proposed a method using a “reference marker” next to the wound with a segmentation process carried out by their Swift Wound app, based on an undisclosed deep learning model. They got high inter-rater reliabilities (ICC = 0.97–1.00). Kompalliy et al.^36^ proposed a method using a “scale” beside the wound and segmentation software by which the user marks the outside and inside of the wound. In the review article of Lucas et al.^37^, the author suggested combining an add-on spatial sensor and machine learning for segmentation as future directions.

In our study, the automatic area measurement by LiDAR technology and the U-Net model, after deleting the two outliers, had acceptable accuracy and precision, with a 17.7% MRE and 0.125 SD. Most importantly, our system was built by LiDAR technology and a state-of-art deep learning segmentation model so that all that is needed to use it is a high-level smartphone or tablet (iPhone 12 Pro or iPad Pro or more advanced types) with the App we built, no additional device such as an add-on infrared 3D sensor and no additional movement of the fingertips to mark the wound contour. It is a fully “automatic” area measuring tool offering convenience and efficiency. To our knowledge, our study is the first study utilizing LiDAR technology for wound measurement.

### Limitations

The main limitation of this study is with limited photos. On the segmentation and automatic area measurement task, limited training data did result in overfitting by Mask-RCNN but for U-Net the limited number of training photos was adequate. These results informed us that we need more training photos for Mask R-CNN than for U-Net. A large prospective clinical study and users’ feedback would be necessary to further verify the effectiveness.

Another issue is that our photos could not reveal drainage sinus and deep dead space especially when they were taken by nonprofessional first-line caregivers. In a deep wound, some parts of the wound may appear dark in the picture. Thus, it may be advisable to make a note in our system that deep wounds, especially with draining sinuses or tunneling, may be incorrectly measured.

### Strengths

Although this is not the first study to utilize deep learning for segmentation of pressure injuries, it is the first study to combine 3D imaging technology and a deep learning segmentation model. Most importantly, our 3D imaging applied LiDAR technology, which is implanted in high-level smartphones and tablets such as the iPhone 12 Pro, iPad Pro and more advanced types, without requiring additional devices such as a label card or a ruler beside the wound or an add-on infrared 3D sensor mounted on the camera. Such convenience and efficiency would make clinical application more feasible.

## Conclusion

For automatic wound segmentation, the performance of the U-Net model with a ResNet-101 backbone was better than Mask R-CNN with a ResNet-101 backbone. For small and limited dataset, U-Net is an appropriate model for segmentation of biomedical images.

For automatic wound area measurement, we combined LiDAR technology and a previously trained segmentation model, U-Net with a ResNet-101 backbone, and obtained acceptable results in our prospective clinical study.

## Supplementary Information

Supplementary Video legend.

Supplementary Video 1.

Supplementary Figure S1.

Supplementary Figure S2.

Supplementary Figure S3.

Supplementary Figure S4.

Supplementary Figure S5.

Supplementary figure legends.

## Data Availability

The datasets generated and/or analyzed during the current study are available in the https://drive.google.com/drive/folders/15T2BBlxdYPpUKhXE7lrRdnIffYM0Nj_9. The data that support the findings of this study are available from National Taiwan University but restrictions apply to the availability of these data, which were used under license for the current study, and so are not publicly available. Data are however available from the authors upon reasonable request and with permission of National Taiwan University.
