# Supplementary material for: Automatic segmentation and measurement of pressure injuries using deep learning models and a LiDAR camera
Source: Sci Rep. 2023 Jan 13;13:680. doi: 10.1038/s41598-022-26812-9 (PMC9839689; doi:10.1038/s41598-022-26812-9)
Supplement: Supplementary file 1 — Supplementary Video legend. [file 41598_2022_26812_MOESM1_ESM.docx]

Supplementary video title and legend

Video S1. The demonstration of the automatic area measurement system of pressure injuries.

We create an application named “Pressure Ulcer Measure”. Everyone can download this application to measure the area of the pressure injuries via Apple Store. But be aware that this application can only run on the iOS/iPadOS device with LiDAR. After taking the photo of the pressure injury, just click "SEGMENT" button of application. It will do auto segmentation and area measurement.
