# Supplementary material for: Automatic segmentation and measurement of pressure injuries using deep learning models and a LiDAR camera
Source: Sci Rep. 2023 Jan 13;13:680. doi: 10.1038/s41598-022-26812-9 (PMC9839689; doi:10.1038/s41598-022-26812-9)
Supplement: Supplementary file 8 — Supplementary figure legends. [file 41598_2022_26812_MOESM8_ESM.docx]

Supplementary files

Figure S1. U-Net architecture.

Figure S2. Mask R-CNN architecture

Figure S3. The regression plane

Figure S4. Demonstration 1 of the segmentation by U-Net and Mask R-CNN. Left: original image. Upper middle: image resized to 512*512. Upper right: ground truth. Lower middle: result of U-Net. Lower right: result of Mask R-CNN.

Figure S5. Demonstration 2 of the segmentation by U-Net and Mask R-CNN.

Left: original image. Upper middle: image resized to 512*512. Upper right: ground truth. Lower middle: result of U-Net. Lower right: result of Mask R-CNN.
